# Outcomes of Advanta V12 Covered Stents After Fenestrated Endovascular Aneurysm Repair

**DOI:** 10.1177/15266028211016423

**Published:** 2021-05-19

**Authors:** Claire van der Riet, Richte C. L. Schuurmann, Eric L. G. Verhoeven, Clark J. Zeebregts, Ignace F. J. Tielliu, Reinoud P. H. Bokkers, Athanasios Katsargyris, Jean-Paul P. M. de Vries

**Affiliations:** 1 Department of Surgery, Division of Vascular Surgery, University Medical Center Groningen, University of Groningen, Groningen, the Netherlands; 2 Multimodality Medical Imaging Group, Technical Medical Centre, University of Twente, Enschede, the Netherlands; 3 Department of Vascular and Endovascular Surgery, General Hospital Nuremberg, Paracelsus Medical University, Nuremberg, Germany; 4 Department of Radiology, Medical Imaging Center, University Medical Center Groningen, University of Groningen, Groningen, the Netherlands

**Keywords:** abdominal aortic aneurysm, endograft, endovascular aortic aneurysm repair, balloon-expandable covered stent, stent-graft

## Abstract

**Purpose:**

Fenestrated endovascular aneurysm repair (FEVAR) is a well-established endovascular treatment option for pararenal abdominal aortic aneurysms in which balloon-expandable covered stents (BECS) are used to bridge the fenestration to the target vessels. This study presents midterm clinical outcomes and patency rates of the Advanta V12 BECS used as a bridging stent.

**Methods:**

All patients treated with FEVAR with at least 1 Advanta V12 BECS were included from 2 large-volume vascular centers between January 2012 and December 2015. Primary endpoints were freedom from all-cause reintervention, and freedom from BECS-associated complications and reintervention. BECS-associated complications included significant stenosis, occlusion, type 3 endoleak, or stent fracture. Secondary endpoints included all-cause mortality in-hospital and during follow-up.

**Results:**

This retrospective study included 194 FEVAR patients with a mean age of 72.2±8.0 years. A total of 457 visceral arteries were stented with an Advanta V12 BECS. Median (interquartile range) follow-up time was 24.6 (1.6, 49.9) months. The FEVAR procedure was technically successful in 93% of the patients. Five patients (3%) died in-hospital. Patient survival was 77% (95% CI 69% to 84%) at 3 years. Freedom from all-cause reintervention was 70% (95% CI 61% to 78%) at 3 years, and 33% of all-cause reinterventions were BECS associated. Complications were seen in 24 of 457 Advanta V12 BECSs: type 3 endoleak in 8 BECSs, significant stenosis in 4 BECSs, occlusion in 6 BECSs, and stent fractures in 3 BECSs. A combination of complications occurred in 3 BECSs: type 3 endoleak and stenosis, stent fracture and stenosis, and stent fracture and occlusion. The freedom from BECS-associated complications for Advanta V12 BECSs was 98% (95% CI 96% to 99%) at 1 year and 92% (95% CI 88% to 95%) at 3 years. The freedom from BECS-associated reinterventions was 98% (95% CI 95% to 100%) at 1 year and 94% (95% CI 91% to 97%) at 3 years.

**Conclusion:**

The Advanta V12 BECS used as bridging stent in FEVAR showed low complication and reintervention rates at 3 years. A substantial number of FEVAR patients required a reintervention, but most were not BECS related.

## Introduction

Fenestrated endovascular aneurysm repair (FEVAR) for the treatment of pararenal abdominal aortic aneurysms (pAAAs) is associated with high technical success (97%), low intraoperative visceral artery occlusion rates (1%), and low 30-day mortality (1%).^1^ However, within 3 to 5 years after FEVAR, reinterventions have been reported in 27% to 42% of patients.^2^ In 28%, this is due to target vessel–associated endoleaks and in 11% to 13% to stenosis or occlusion of visceral arteries. These complications are frequently reported to be caused by fenestration misalignment and stent disintegrity.^3,4^

The Advanta V12 (Atrium Medical Corporation, Merrimack, NH, USA) balloon-expandable covered stent (BECS) is a stainless steel stent with open cells, encapsulated in polytetrafluoroethylene and premounted on a noncompliant balloon catheter. This BECS is still used in an off-label way to bridge the fenestration of the fenestrated stent-graft (FSG) to the target vessel in FEVAR procedures. To date, there are no BECS that can be used on-label for this indication. A study with the Bentley BeGraft (Bentley Innomed, Heckingen, Germany) is on its way, but results are awaited. Literature about BECS patency rates is substantial, but most of the studies reported the short-term results based on a mixture of BECSs.^5^ This retrospective study specifically focuses on the midterm clinical outcomes and patency rates of the Advanta V12 BECS used as a bridging stent.

## Materials and Methods

### Study Design and Population

This is a retrospective study of 2 large-volume FEVAR center cohorts of the General Hospital, Nuremberg, Germany, and the University Medical Center Groningen, The Netherlands. Patients consecutively underwent elective FEVAR for a pAAA between January 2012 and December 2015. The following inclusion criteria were defined: patients treated with FEVAR to treat a pAAA as a first treatment or suprarenal fenestrated extension to treat a previous failed EVAR, and at least 1 fenestration bridged with an Advanta V12 BECS.

The respective Medical Ethical Institutional Review Boards granted dispensation for the study from the Medical Research Involving Human Subjects Act (WMO) obligation (registration number: 2019/00562). As a consequence, informed consent was not obtained. Patient data were processed and electronically stored in agreement with the Declaration of Helsinki’s ethical principles for medical research involving human subjects. Data were stored and analyzed anonymously.

### Endpoints

Clinical data, as available until November 2019, were retrospectively collected from the electronic patient records and registered in a Research Electronic Data Capture (REDCap, version 8.10.18; Vanderbilt University, Nashville, TN, USA). Technical success was defined as successful deployment of the planned FSG and BECS(s) with patent target vessel(s) and absence of type 1 or 3 endoleak on completion angiography. Primary endpoints were freedom from all-cause reintervention and freedom from BECS-associated complication and reintervention. BECS-associated complications were defined as significant stenosis (>50%) on duplex ultrasonography (DUS) or computed tomography angiography (CTA), occlusion, type 3 endoleak, or stent fracture. Stent fractures were not subclassified. The complications were registered according to the reports of the CTA scans with ≤1.5-mm slice thickness and axial and coronal/sagittal reconstructions or DUS. The regular follow-up protocol included a CTA scan at 30 days and at 1 year post-FEVAR, followed by yearly CT scan, or DUS combined with X-ray. Only in case of complications or clinical complaints unplanned imaging was performed. All CTA and DUS imaging examinations were analyzed. Secondary endpoints included all-cause mortality, in-hospital and during follow-up.

### Statistical Analysis

Data were analyzed using SPSS 23 statistical software (IBM Corp, Armonk, NY, USA). Normality of the data was assessed via visual inspection of Q-Q plots. Normally distributed variables were expressed as mean ± SD and nonnormally distributed variables were expressed as median (interquartile range). Kaplan-Meier analyses were performed to estimate patient survival, freedom from all-cause reintervention, and freedom from BECS-associated complications and BECS-associated reinterventions. The difference in freedom from BECS-associated complications between the renal arteries was tested by a log-rank test. p values were considered statistically significant when the 2-tailed α was <0.05.

## Results

### Study Population

This study included 194 patients (mean age, 72.2±8.0 years; 84% male) treated by FEVAR and at least 1 Advanta V12 BECS, comprising 142 patients from the General Hospital Nuremberg and 52 patients from the University Medical Center Groningen. The patient demographics are summarized in Table 1. FEVAR was used in 177 patients (91%) to treat a pAAA as a first treatment, and 17 patients (9%) were treated with a fenestrated cuff for a type 1a endoleak after previous EVAR. A Zenith FSG (Cook Medical Inc, Bloomington, IN, USA) was used in 187 patients (96%), and an Anaconda FSG (Terumo Aortic, Inchinnan, Scotland, UK) was used in 7 patients (4%). Patients had a median of 2 (1, 4) CTA scans post-FEVAR, with a median duration of CTA follow-up of 22.4 (1.2, 48.7) months. The median duration of overall CTA or DUS surveillance was 24.6 (1.6, 49.9) months.

**Table 1.** Patient Demographics (n=194).^a^

Variable	Mean ± SD or n (%)
Age (years)	72.2±8.0
Male sex	162 (84)
eGFR (mL/min)	61.0±20
Hypertension [systolic blood pressure >140 mm Hg]	160 (82)
Diabetes mellitus	27 (14)
Coronary artery disease	104 (54)
COPD	80 (41)
Pre-FEVAR aneurysm diameter (mm)	59.8±10.3
ASA physical status ≥III:	95 (49)

Abbreviations: ASA, American Society of Anesthesiologists; COPD, chronic obstructive pulmonary disease; eGFR, estimated glomerular filtration rate; FEVAR, fenestrated endovascular aneurysm repair.

a Categorical data are presented as n (%); continuous data are presented as mean ± SD.

The study population included 15, 92, 72, and 15 patients who were treated with a 1, 2, 3, and 4 FSG configurations, respectively. These patients had, in total, 457 fenestrations stented with an Advanta V12 BECS: 174 left renal arteries (LRAs), 179 right renal arteries (RRAs), 90 superior mesenteric arteries (SMAs), and 14 celiac trunks. The aortic end of the Advanta V12 BECS was flared routinely with an 8×2 or a 10×2 mm balloon depending on the diameter of the BECS. In addition, 18 fenestrations were stented with another type of BECS: 5 BeGraft (Bentley InnoMed, Hechingen, Germany) and 13 Bard Lifestream (Bard Peripheral Vascular Inc, Tempe, AZ, USA). Owing to the low numbers, these were excluded from the current BECS analysis.

### Patient Outcomes

Technical success was achieved in 93% (180 of 194) of the patients. A wire rupture of the renal artery occurred in 1 patient. Five patients had an endoleak at completion angiography (4 type 1a endoleak and 1 type 3 endoleak at the fenestration of the LRA). Three patients had a dissection of a renal artery (2 LRAs and 1 RRA) resulting in occlusion of the artery. A lumbar artery in 1 patient was unintentionally catheterized and stented instead of the RRA, resulting in permanent dialysis. An internal iliac artery was unintentionally covered in 1 patient. In 3 patients, the FEVAR procedure was not completed; in these patients, 2 RRAs and 1 celiac trunk could not be cannulated and stented.

The median intensive care and hospital lengths of stay were 0 (0, 0) and 5 (4, 6) days, respectively. The in-hospital and 30-day mortality was 1.5% (3 of 194) and 2.6% (5 of 194), respectively. Three patients died within 3 days after FEVAR. One patient died of renal bleeding due to wire rupture and bowel ischemia, one patient died of renal failure after an uncompleted procedure, and 1 patient sustained a traumatic subdural hematoma after a fall out of bed. One patient died of multiorgan failure at 62 days and had no post-FEVAR image surveillance. One patient died of acute myocardial infarction at 101 days and showed no BECS-related complications on the first post-FEVAR CTA scan.

**Figure 1.**
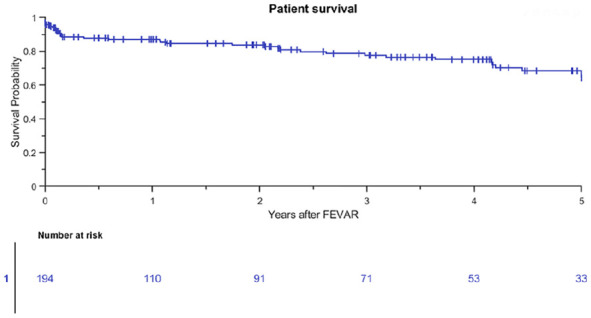
Kaplan-Meier patient survival (standard error 4.8% at 4- to 5-year interval). FEVAR, fenestrated endovascular aneurysm repair.

**Figure 2.**
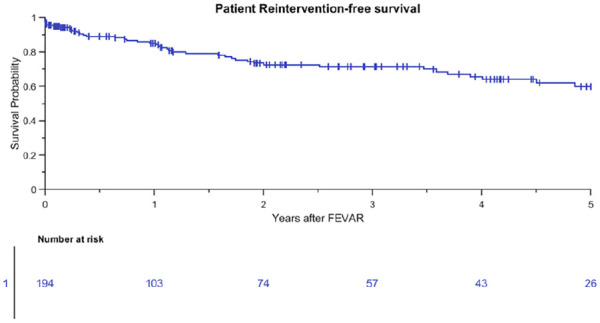
Kaplan-Meier curve for freedom from all-cause reintervention of patients (standard error 5.3% at 4- to 5-year interval). FEVAR, fenestrated endovascular aneurysm repair.

The study population included 7, 32, 28, and 4 patients who were treated with a 1, 2, 3, and 4 FSG configuration at 3 years, respectively. Estimated patient survival was 77% (95% CI 69% to 84%) at 3 years (Figure 1). Freedom from all-cause reintervention was 70% (95% CI 61% to 78%) at 3 years, and 33% of all-cause reinterventions were BECS associated (Figure 2). A total of 91 patients with 198 Advanta V12 BECSs were lost to follow-up at 1 year; 42 patients underwent follow-up at other hospitals in other regions of the respective countries, 21 patients underwent a reintervention, 14 patients did not show up for follow-up and did not respond to repeated calls, and 14 patients died during the first year after the FEVAR procedure. The indications for the first post-FEVAR reinterventions are summarized in Table 2. Eight patients underwent multiple reinterventions.

**Table 2.** Indication for First Reintervention.

Variable	Patients (n=43)
Rupture due to	
Type 1b endoleak	1
Type 2 endoleak	1
Type 3c endoleak	1
Endoleak	
Type 1a	3^a^
Type 1b	2
Type 1b and 3b	1
Type 1b and type 3c (LRA)	1
Type 1c (LRA)	1
Type 2	9
Type 2 and type 3c (LRA)	1
Type 3c (1× LRA, 2× RRA, 1× celiac trunk)	4
Unspecified endoleak (growing aneurysm sac)	1
Dissection of target vessel (1× LRA, 1× RRA)	2^a^
In BECS stenosis and occlusion	
Stenosis of LRA	1
Stenosis and stent fracture of RRA	1
Occlusion of LRA	1
Occlusion of LRA and stenosis of RRA	1
Complications iliac trajectory	
Stent fracture	2
Dislocation	1
Dissection of external iliac artery	1^a^
Bleeding	
External iliac artery	2^b^
Left kidney	2^b^
Complications of nonstented visceral arteries in a scallop (1× SMA, 1× celiac trunk)	2
Fenestrated stent graft infection	1

Abbreviations: BECS, balloon-expandable covered stent; LRA, left renal artery; RRA, right renal artery; SMA, superior mesenteric artery.

a One reintervention within 30 days.

b Two reinterventions within 30 days.

### BECS Outcomes

**Figure 3.**
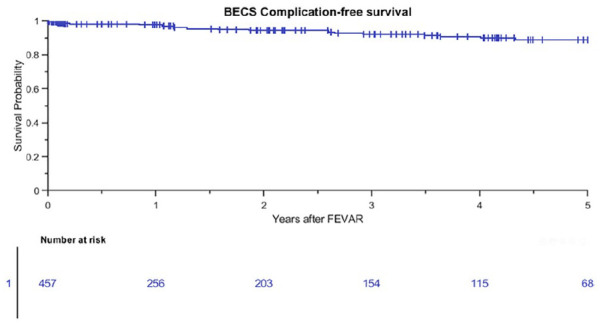
Kaplan-Meier curve for freedom from Advanta V12 balloon-expandable covered stent (BECS)-associated complications (standard error 2.4% at 4- to 5-year interval). FEVAR, fenestrated endovascular aneurysm repair.

**Figure 4.**
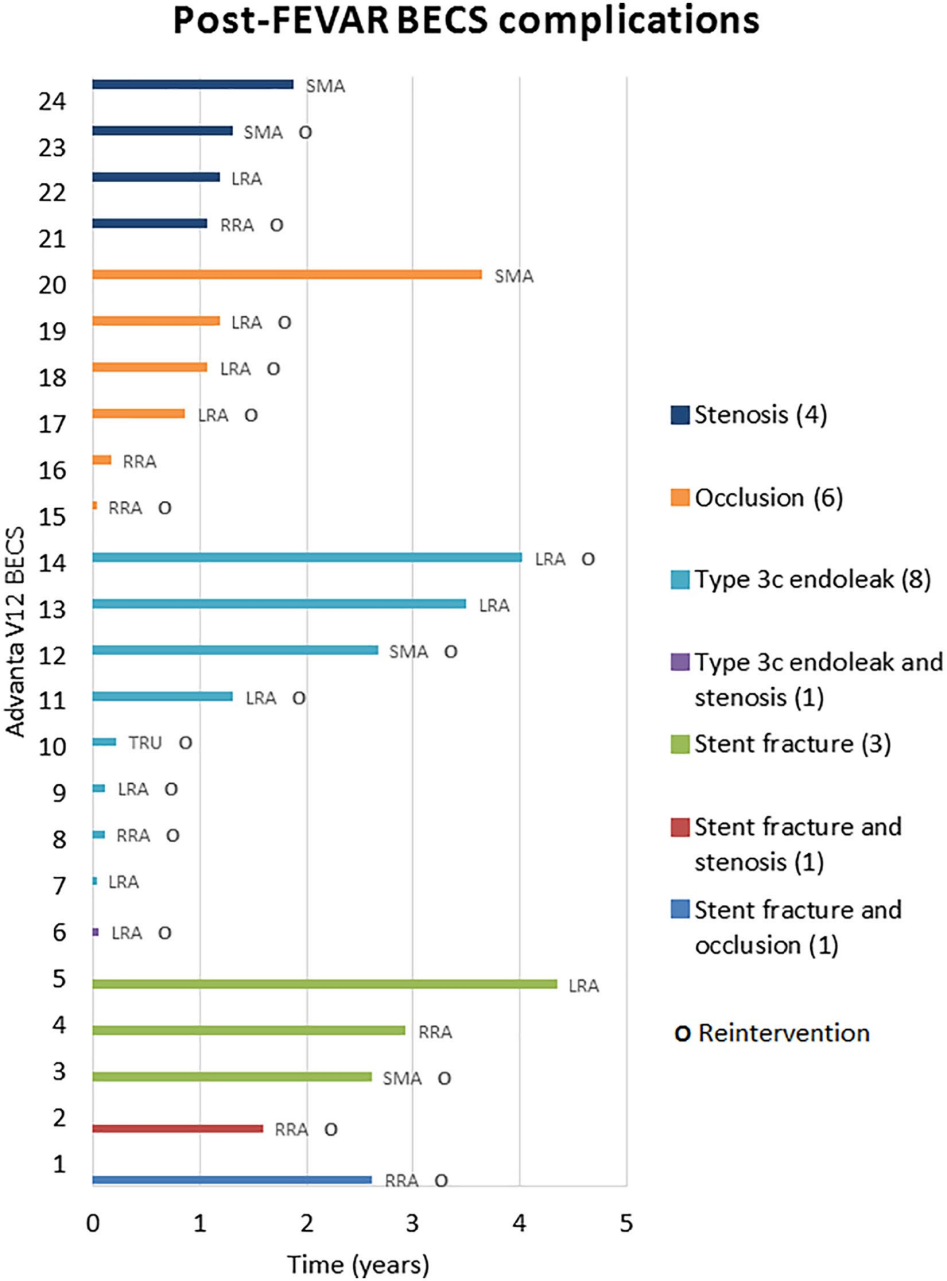
Occurrence of postoperative balloon-expandable covered stent (BECS) complications over time (years). FEVAR, fenestrated endovascular aneurysm repair; LRA, left renal artery; RRA, right renal artery; SMA, superior mesenteric artery; TRU, celiac trunk.

**Figure 5.**
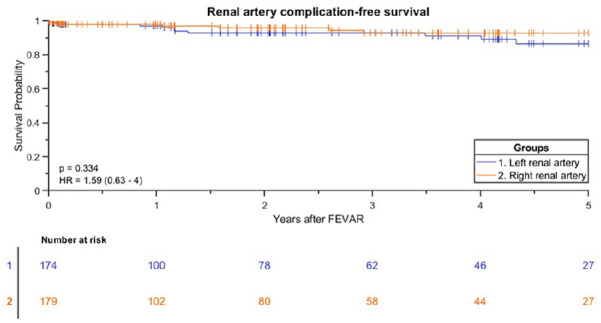
Kaplan-Meier curve for freedom from Advanta V12 balloon-expandable covered stent-associated complication for the left and right renal artery (standard error 4.3% and 2.8% at 4- to 5-year interval, respectively). FEVAR, fenestrated endovascular aneurysm repair.

The Kaplan-Meier freedom from Advanta V12 BECS-associated complications was 98% (95% CI 96% to 99%) at 1 year and 92% (95% CI 88% to 95%) at 3 years (Figure 3). Freedom from Advanta V12 BECS-associated reinterventions was 98% (95% CI 95% to 100%) at 1 year and 94% (95% CI 91% to 97%) at 3 years. There were 24 Advanta V12 BECS-associated complications reported: 11 of the 174 (6%) LRA BECS, 7 of the 179 (4%) RRA BECS, 5 of the 90 (6%) SMA BECS, and 1 of the 14 (7%) celiac trunk BECS. The median BECS diameter was not different between the BECS group with (n=24) and without (n=433) complicated follow-up. For both groups, the median BECS diameter was 6.0 (6.0, 7.0) mm. Figure 4 depicts the time interval from FEVAR up to diagnosis of the first complication and of the reintervention. Eight BECSs showed a type 3 cendoleak, and 6 needed a reintervention. Six BECSs occluded, and 4 underwent a reintervention. A significant stenosis was reported in 4 BECSs, and 2 underwent a reintervention. Three BECSs fractured, and 1 underwent a reintervention. In addition, a combination of complications occurred in 3 BECSs: type 3c endoleak and stenosis, stent fracture and stenosis, and stent fracture and occlusion. All 3 BECSs needed a reintervention. In total, 16 of 24 (67%) BECSs with a complication underwent a reintervention, and this was successful in 12 cases. The freedom from a BECS-associated complication was not significantly different (p=0.334) for the side of the renal arteries: 93% (95% CI 85% to 97%) for the RRA and 91% (95% CI 83% to 95%) for the LRA at 3 years (Figure 5).

## Discussion

This study shows the midterm results of the Advanta V12 BECS used as a bridging stent in FEVAR. Although a substantial number of patients needed post-FEVAR reinterventions, one-third were due to BECS-associated complications. Previous publications reported technical success and short-term target vessel patency of 96% to 98%, which is similar to our results.^5–7^ Contrary to the current study, these numbers were reported for a mixture of BECSs and also included branched endovascular aortic repairs.

Concerning the substantial need for all-cause reinterventions, open surgical repair may still be the preferred treatment for patients at good surgical risk.^8,9^ For frail patients, however, FEVAR provides a durable solution at the trade-off of the reported reintervention rates. Most reinterventions can be performed by endovascular means.

BECS-associated complications can have different causes that may need different treatments. A mismatch of the diameter of the BECS with the target vessel or the fenestration can result in loss of fixation and seal.^10^ Increased length of the flared part of the BECS to provide stability and seal comes, however, at the cost of increased risk for stenosis and occlusion.^11^ Too much oversizing of the distal end of the BECS in the visceral arteries may increase the risk for dissections and dissection-associated occlusions.

A second potential cause for complications is caudal migration of the FSG with displacement of the BECS. This can result in stent fracture and compression of the BECS between the fenestration and the target vessel, potentially occluding the target vessel. England et al^12^ reported median (interquartile range) caudal migration of 6.0 (4.1, 10.0) mm in 23% of FEVAR patients at 3 years of follow-up. BECS displacement caused by continuous respiratory and hemodynamic motions may also result in a type 3 endoleak between the fenestration and the BECS.^13^ The LRA is more affected by respiratory motion than the RRA, and therefore BECSs in the LRA may be more susceptible to complications.^14^ The current study, however, showed no significant difference in BECS-associated complications between the renal arteries.

Differentiation between these underlying causes on X-ray imaging and CT scans is essential to choose and plan the right reintervention. It can, however, be difficult to visualize the BECS in detail or determine the exact origin of an endoleak due to artifacts of the FSG and BECS on the CTA scan. Moreover, small changes in FSG or BECS geometry at consecutive follow-up CTA scans may be missed with the current scan protocols. Geometric analysis of the BECS and detection of subtle displacement of the stent over time with dedicated imaging software could improve correct differentiation of the underlying cause of a complication and may even predict it.^15^ The importance of frequent post-FEVAR image surveillance to detect changes of the BECS configuration has been suggested in a previous study.^16^ In addition to CT, X-ray imaging can be used to inspect stent integrity and DUS can provide information about blood flow profiles in the visceral arteries. These imaging methods should be combined during follow-up, especially in patients with AAA growth or endoleaks post-FEVAR.

One of the limitations of this study is its retrospective design. Second, a substantial part of the study population was lost to follow-up due to several reasons. Moreover, the causes of death were not available for all patients, so only all-cause mortality was reported. Third, the current study included a substantial number of 1 and 2 FSG configurations, which was more common during the inclusion period (2012–2015) than it is today. Nowadays, FEVAR with 3 and 4 FSG configurations are preferred to secure a longer seal zone.^17^ On the other hand, patients are still being treated by 2-FEVAR and a scallop for the SMA, which is a good alternative for open surgery or chimney EVAR procedures for juxtarenal AAAs. The inclusion period was chosen to enable assessment of at least 3 years of follow-up. The difference in BECS-associated complications between the renal arteries was studied, but the SMA and celiac trunk were excluded from this subanalysis because the numbers were too small.

## Conclusion

The Advanta V12 BECS used as bridging stent in FEVAR showed low complication and reintervention rates at 3 years. A substantial number of FEVAR patients required a reintervention, but most were not BECS related.

## References

[1] Verhoeven EL Katsargyris A Oikonomou K, et al. Fenestrated endovascular aortic aneurysm repair as a first line treatment option to treat short necked, juxtarenal, and suprarenal aneurysms. Eur J Vasc Endovasc Surg. 2016;51:775–781. 26860255 10.1016/j.ejvs.2015.12.014

[2] Dossabhoy SS Simons JP Diamond KR, et al. Reinterventions after fenestrated or branched endovascular aortic aneurysm repair. J Vasc Surg. 2018;68:669–681. 29523438 10.1016/j.jvs.2017.12.036

[3] Crawford SA Osman E Doyle MG, et al. Impact of fenestrated stent graft misalignment on patient outcomes. J Vasc Surg. 2019;70:1056–1064. 30928171 10.1016/j.jvs.2018.12.047

[4] Gibello L Ruffino MA Varetto G, et al. Current results of balloon expandable visceral stent-grafts in fenestrated endografting. J Cardiovasc Surg (Torino). 2020;61:37–46. 10.23736/S0021-9509.19.11199-8 31815375

[5] Mezzetto L Scorsone L Silingardi R, et al. Bridging stents in fenestrated and branched endovascular aneurysm repair: a systematic review. Ann Vasc Surg. Published online January 5, 2021. doi:10.1016/j.avsg.2020.10.052 33359330

[6] Spear R Sobocinski J Hertault A, et al. One year outcomes of 101 BeGraft stent grafts used as bridging stents in fenestrated endovascular repairs. Eur J Vasc Endovasc Surg. 2018;55:504–510. 29501401 10.1016/j.ejvs.2018.01.023

[7] Motta F Parodi FE Knowles M, et al. Performance of Viabahn balloon-expandable stent compared with self-expandable covered stents for branched endovascular aortic repair. J Vasc Surg. 2021;73:410–416.e2. 32473341 10.1016/j.jvs.2020.05.028

[8] Beach JM Rajeswaran J Parodi FE, et al. Survival affects decision making for fenestrated and branched endovascular aortic repair. J Vasc Surg. 2018;67:722–734.e8. 28967527 10.1016/j.jvs.2017.07.118

[9] Jones AD Waduud MA Walker P, et al. Meta-analysis of fenestrated endovascular aneurysm repair versus open surgical repair of juxtarenal abdominal aortic aneurysms over the last 10 years. BJS Open. 2019;3:572–584. 31592091 10.1002/bjs5.50178 PMC6773647

[10] Oshin OA How TV Brennan JA, et al. Magnitude of the forces acting on target vessel stents as a result of a mismatch between native aortic anatomy and fenestrated stent-grafts. J Endovasc Ther. 2011;18:569–575. 21861749 10.1583/11-3471.1

[11] Kandail H Hamady M Xu XY Effect of a flared renal stent on the performance of fenestrated stent-grafts at rest and exercise conditions. J Endovasc Ther. 2016;23:809–820. 27225213 10.1177/1526602816651425 PMC5023035

[12] England A Garcia-Finana M McWilliams RG Multicenter retrospective investigation into migration of fenestrated aortic stent grafts. J Vasc Surg. 2015;62:884–892. 26213272 10.1016/j.jvs.2015.04.420

[13] Kärkkäinen J Tenorio E Jain A, et al. Outcomes of target vessel endoleaks after fenestrated-branched endovascular aortic repair. J Vasc Surg. 2020;72:445–455. 31980247 10.1016/j.jvs.2019.09.055

[14] Suh GY Choi G Herfkens RJ, et al. Three-dimensional modeling analysis of visceral arteries and kidneys during respiration. Ann Vasc Surg. 2016;34:250–260. 27116907 10.1016/j.avsg.2016.04.004 PMC4930742

[15] Overeem S Schuurmann R Schumacher M, et al. Validation of a novel methodology to evaluate changes in the flare geometry of renovisceral bridging stent-grafts after fenestrated endovascular aneurysm repair. J Endovasc Ther. 2020;27:436–444 32314657 10.1177/1526602820915932

[16] de Niet A Post RB Reijnen M, et al. Geometric changes over time in bridging stents after branched and fenestrated endovascular repair for thoracoabdominal aneurysm. J Vasc Surg. 2019;70:702–709. 30837180 10.1016/j.jvs.2018.12.023

[17] Katsargyris A Oikonomou K Kouvelos G, et al. Comparison of outcomes for double fenestrated endovascular aneurysm repair versus triple or quadruple fenestrated endovascular aneurysm repair in the treatment of complex abdominal aortic aneurysms. J Vasc Surg. 2017;66:29–36. 28189357 10.1016/j.jvs.2016.11.043

